# Transformation of 5-D itch scale and numerical rating scale in chronic hemodialysis patients

**DOI:** 10.1186/s12882-017-0475-z

**Published:** 2017-02-08

**Authors:** Jia-Wen Lai, Hung-Chih Chen, Che-Yi Chou, Hung-Rong Yen, Tsai-Chung Li, Mao-Feng Sun, Hen-Hong Chang, Chiu-Ching Huang, Fuu-Jen Tsai, Johannes Tschen, Chiz-Tzung Chang

**Affiliations:** 1 College of Life Sciences, National Chung Hsing University, Taichung, Taiwan; 2 Kidney Institute and Division of Nephrology, China Medical University Hospital, No. 2, Yu-der Road, North District, Taichung, 40447 Taiwan; 3 College of Medicine, China Medical University, Taichung, Taiwan; 4 Department of Internal Medicine, Division of Nephrology, Asia University Hospital, Wufeng, Taichung Taiwan; 5 Department of Biotechnology, Asia University, Wufeng, Taichung Taiwan; 6 Department of Chinese Medicine, China Medical University Hospital, Taichung, Taiwan; 7 School of Chinese, China Medical University, Taichung, Taiwan; 8 Research Center for Chinese Medicine & Acupuncture, China Medical University, Taichung, Taiwan; 9 Research Center for Traditional Chinese Medicine, China Medical University, Taichung, Taiwan; 10 Graduate Institute of Biostatistics, China Medical University, Taichung, Taiwan

**Keywords:** 5-D itch scale, Hemodialysis, Pruritus, Numerical rating scale

## Abstract

**Background:**

Pruritus is a common and frustrating symptom in hemodialysis (HD) patients and 5-D itch scale is proposed as a reliable measurement of pruritus. However, information regarding 5-D itch scale categories is currently unavailable. We explored optimal cut-offs 5-D itching scale based on numerical rating scale (NRS) categories in HD patients.

**Methods:**

Four hundred and nine HD patients in China Medical University Hospital in December 2014 were included and severity of pruritus was estimated using NRS and 5-D itch scale. The association of NRS and 5-D itch scale was analyzed by linear regression. The optimal cut-offs for 5-D itch scale based on NRS categories were generated.

**Results:**

The average NRS was 3.4 ± 3.0 and the average 5-D itch scale was 10.9 ± 4.8. The 5-D score was strongly correlated with the NRS: *r* = 0.831 (*p* < 0.001). NRS = −2.31 + 0.52 × (5-D scale). The averages of 5-D scales were 6.4 ± 1.5, 9.6 ± 2.2, 13.1 ± 3.2, 15.7 ± 4.4, 19.5 ± 4.4 for no, mild, moderate, severe, and very severe pruritus based on categorized NRS. A 5-D itch scale categories were proposed, ≤ 8 for NRS = 0, 9–11 for mild pruritus, 12–17 for moderate pruritus, 18–21 for severe pruritus and ≥ 22 for very severe pruritus.

**Conclusions:**

Categories for the 5-D itch scale were proposed based on the measurements of pruritus severity in HD patients. This information provides a simple solution that enables transformation between the 5-D itch scale and the numerical rating scale.

**Electronic supplementary material:**

The online version of this article (doi:10.1186/s12882-017-0475-z) contains supplementary material, which is available to authorized users.

## Background

Pruritus is one of the most common and frustrating symptoms in hemodialysis (HD) patients with a prevalence of approximately 36–50% [1–3]. Pruritus is associated with not only impaired quality of life [4, 5] but also high psychological burden [6]. Because Pruritus is a subjective experience, it is difficult to measure Pruritus objectively. Many assessments of pruritus are currently available, including unidimensional, multidimensional, and other scales (Table 1) but none of them is accepted by most experts as a gold standard. A reliable measurement of pruritus intensity is critical for studies assessing the efficacy of antipruritic treatment [7, 8]. It is generally accepted that at least two different measurements should be used to access pruritus intensity in clinical studies.

**Table 1:** Summary of measurements of pruritus in the literature

Uni-dimensional scale
Visual analogue scale (VAS), numerical rating scale (NRS), and verbal rating scale (VRS)
Multi-dimensional scales
5D itch scale, four-item questionnaire, itch severity scale, pruritus grading system
Measurement of scratching episodes Measurement of limb movements
Imaging of brain activity
Measurement of itch threshold
Scales assessing the psychosocial condition of the patient related to itch ItchQoL, Dermatology Life Quality Index and Beck’s Depression Inventory

Multidimensional scales not only access pruritus intensity but measure the impact of pruritus on patients’ quality of life. Commonly used multidimensional scales include the pruritus grading system [9], the 5-D itch scale [10], and the itch severity scale [11]. The 5-D itch scale was published in 2010 and was validated with Numerical rating scale (NRS) in individuals with human immunodeficiency virus, skin, liver, or kidney disease [10]. The 5-D itch scale, sensitive to the changes of pruritus with time, is a brief, single paged, multidimensional quantification of pruritus. NRS, an unidimensional scale, which may be the most widely used is a validated measurement of UP in HD patients [1, 4, 6]. Pruritus intensity measured using NRS can be categorized into no (0 point), mild (1–3 points), moderate (4–6 points), severe (7–8 points), and very severe pruritus (≥9 points). However, information regarding the 5-D itch scale categories on uremic pruritus is currently not available. The aim of the study is to investigate the 5-D itch scale categories based on NRS categories in HD patients. The information found will enable comparisons of the pruritus intensity and prevalence of pruritus among studies using different score systems. This information would make contribution to bioinformatics researches.

## Methods

All patients who received HD for more than 3 months and signed the inform consent in China Medical University Hospital were enrolled. Patients were excluded if they were younger than 20 years old or were not able to sign their own consent for any reason. The severity of pruritus was measured using a 0-to-10 NRS (0 = no pruritus, 10 = maximal pruritus) [12] and the 5-D itch scale [10]. NRS categories were defined as 0 for no pruritus, 1–3 points for mild pruritus, 4–6 points for moderate pruritus, 7–8 points for severe pruritus, and ≥ 9 points for very severe pruritus [12]. A severity of pruritus more than moderate pruritus was considered as symptomatic pruritus (NRS ≥ 4). The causes of kidney disease were diagnosed by the primary care physician of nephrology at the initiation of HD. Patients’ serum calcium and phosphorus were measured in the same month when the pruritus was measured and their intact parathyroid hormone (iPTH) and Kt/V were measured in 3 months after or before the pruritus was measured.

### Statistical analysis

A descriptive analysis was performed as appropriate: median and interquartile range in the case of non-parametric variable and mean and standard deviation in the case of parametric distributed variable. Analysis of variance, Mann–Whitney *U* test, *t*-test, or chi-square test for categorical variables were performed according to standard indications. The association of 5-D itch scale and NRS was analyzed using linear regression. The receiver operating characteristic curve of the 5-D itch scale in association with symptomatic pruritus (NRS ≥ 4) was generated. All analyses were performed using IBM SPSS Statistics for Windows, Version 22.0 Armonk, NY: IBM Corp and a *p* < 0.05 was considered statistically significant.

## Results

The study population consisted of 409 HD patients with a mean age of 63 ± 13 years (Table 2). The causes of kidney disease include diabetes 45.5%, chronic glomerulonephritis 25.2%, hypertension 18.6% and others 10.3%. The average NRS was 3.4 ± 3.0 and the average 5-D itch scale was 10.9 ± 4.8. The prevalence of uremic pruritus was 43% when uremic pruritus was defined as moderately severe pruritus (NRS ≥ 4). There was no difference in NRS and 5-D itch scale according to the etiology of kidney disease.

**Table 2:** Characteristics of all patients

Characteristics	*N* = 409	
Age (year)	63	±13
Male	215	52.6
Causes of kidney disease		
Diabetes	186	45.0
Chronic glomerulonephritis	103	25.2
Hypertension	78	18.6
Others	42	10.3
Pruritus scale		
Numerical rating scale	3.4	±3.0
5-D itch scale	10.9	±4.8
Calcium (mg/dl)	9.3	±0.9
Phosphorus (mg/dl)	5.6	±1.5
iPTH (pg/ml)	111.1	448.9
Kt/V	1.3	±0.3

*iPTH* intact parathyroid hormone in interquartile range

**Fig. 1:**
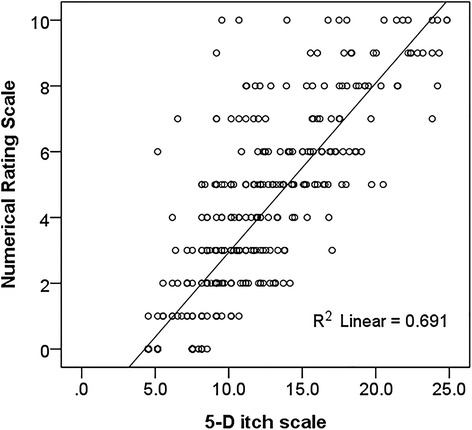
Scatter plot of 5-D itch and numerical rating scale (NRS) with regression line

**Fig. 2:**
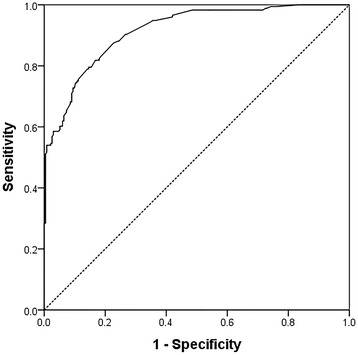
The area under the receiver operating characteristic curve of 5-D itch scale for symptomatic pruritus defined as numerical rating scale ≥ 4

The 5-D score was strongly correlated with the NRS and the Pearson’s correlation coefficients were *r* = 0.831 (*p* < 0.001). An equation: NRS = −2.31 + 0.52 × (5-D itch scale) was generated with a R^2^ of 0.684 (Fig. 1). The area under the receiver operating characteristic curve (Fig. 2) of 5-D itch scale was 0.915 (95% confidence interval: 0.889–0.941, *p* < 0.001) for moderately severe pruritus defined by NRS ≥ 4.

Based on the NRS categories, 100 (24.4%) patients had NRS = 0, 176 (43%) patients had moderate to very severe pruritus (NRS ≥ 4), 133 (32.5%) patients had mild pruritus (NRS 1–3), 107 (26.2%) patients had moderate pruritus (NRS 4–6), 39 (9.5%) patients had severe pruritus (NRS 7–8), and 30 (7.3%) patients had very severe pruritus (NRS ≥ 9, Table 3). The 5-D itch scale was 6.4 ± 1.5 in patients with NRS = 0, 9.6 ± 2.2 in patients with mild pruritus, 13.1 ± 3.2 in patients with moderate pruritus, 15.7 ± 4.4 in patients with severe pruritus, and 19.5 ± 4.4 in patients with very severe pruritus. The minimal-maximal 5-D itch scales were 5–9, 5–17, 7–21, 9–25, 5–25 in patients with NRS = 0, mild, moderate, severe, and very severe pruritus, respectively.

**Table 3:** Statistics of 5-D itch scale according to numerical rating scale (NRS)

NRS categories	5-D itch scale			
	n	Mean ± SD	Min	Max
No pruritus (0)	100	6.4 ± 1.5	5	9
Mild (1–3)	133	9.6 ± 2.2	5	17
Moderate (4–6)	107	13.1 ± 3.2	7	21
Severe (7–8)	39	15.7 ± 4.4	9	25
Very severe (≥9)	30	19.5 ± 4.4	5	25

*NRS* numerical rating scale

By applying the equation: NRS = −2.31 + 0.52 × (5-D itch scale), we propose the following 5-D itch scale categories: ≤ 8 for NRS = 0, 9–11 for mild pruritus, 12–17 for moderate pruritus, 18–21 for severe pruritus, and ≥ 22 for very severe pruritus (Table 4). Using the cut-offs, 262 (64.1%) patients were classified as the same severity categories in the 5-D itch scale categories and in the NRS categories (gray area). 38 (9.3%) patients were classified as more severe pruritus categories (dark gray area) and 108 (26.4%) patients were classified as less severe pruritus categories in the 5-D categories (light gray area). The prevalence of pruritus was 38.4% if an cut-off 12 was used to defined moderate to very severe pruritus. When NRS ≥ 4 was used as a standard to define pruritus, the sensitivity and specificity were 75% and 89.3% for 5-D itch scale with 12 as a cut-off. We did not find significant association between 5-D itch scale and serum calcium (*p* = 0.09), phosphorus (*p* = 0.12), Kt/V (*p* = 0.31), and iPTH (log-transformed, *p* = 0.47).

**Table 4:** Categories of 5-D itch scale compared to numerical rating scale categories

*n* (%)		Numerical rating scale				
		No pruritus (0) *n* = 100	Mild (1–3) *n* = 133	Moderate (4–6) *n* = 106	Severe (7–8) *n* = 39	Very severe (≥9) *n* = 30
5-D itch scale	≤8 n = 141	99 (70.2)	36 (25.5)	5 (3.5)	1 (0.7)	0 (0)
	9–11 n = 110	1 (0.9)	72 (65.5)	27 (24.5)	7 (6.4)	3 (2.7)
	12–17 n = 109	0 (0)	25 (22.9)	65 (59.6)	15 (13.8)	4 (3.7)
	18–21 n = 32	0 (0)	0 (0)	9 (28.1)	13 (40.6)	10 (31.3)
	≥22 n = 16	0 (0)	0 (0)	0 (0)	3 (18.8)	13 (81.2)

## Discussion

In this cross-sectional study, we purposed 5-D itch scale categories based on their correlation with NRS categories of pruritus in HD patients. Information obtained from this study may enable an easy transformation between 5-D itch scale and NRS. The prevalence of pruritus is 43% using NRS (≥4) in this population and this prevalence is similar to that reported in the previous studies [2, 13]. The prevalence of pruritus is slightly lower (38%) when the 5-D itch scale with a cut-off of 12 points is applied. There is no truly “no pruritus” in the 5-D itch scale and the 5-D itch scale is 7.5 points higher than the NRS. A 5-D itch scale less than 8 points may be equal to NRS = 0. In addition, the prevalence of pruritus is lower when a cut-off of 12 points is used in the 5-D itch scale. A cut-off of 10 points of 5-D itch scale results in a close percentage of patients with pruritus defined by NRS ≥ 4. As shown in Table 3, 26.4% patients have higher pruritus intensity using NRS but have lower pruritus intensity in the 5-D itch scale. This might suggest over-estimation of pruritus in NRS. The NRS can be obtained in 10 s and this is the major advantage of NRS. It usually took one to two minutes to complete the 5-D questionnaire in most patients. The upper arm and forearm are the most common locations of pruritus in HD patients and most of the pruritus the tape used during HD. There are good correlations (0.88–0.92) between NRS and each domain of the 5-D itch scale. The 5-D itch scale provided detailed assessments on the distribution of pruritus and the effect of pruritus on quality of life. Some patients may report a higher NRS when they have the 5-D questionnaire before NRS but this difference is not statistically significant.

We also investigated factors associated with uremic pruritus, but we did not find a significant association of pruritus with patients’ age, gender, causes of kidney disease, calcium, phosphorous, Kt/v, and iPTH. Prior to the study, we suspect that patients with diabetes as the primary kidney disease may be associated with pruritus. The prevalence of uremic pruritus (44%) in patients with diabetes as their primary kidney disease was similar to the prevalence (42.2%) of non-diabetic patients. Diabetes was not associated with higher NRS and 5-D itch scale in linear regression, either.

There were some potential limitations of this study. First, the cut-offs of 5-D itch scale were determined using the equation generated from linear regression. Validation of the sensitivity and specificity using a different set of data is needed in order to improve the generalizability of this finding. Second, the prevalence of uremic pruritus may be may be more prevalent in cold seasons [14]. As this study was conducted in December, the prevalence of uremic pruritus may be relatively high. Third, the severity of pruritus may be under-estimated using the 5-D itch scale categories when compared to NRS categories (Table 3). As there is no gold standard measurement of pruritus available, we were not able to identify which measurements of pruritus was more reliable.

## Conclusions

The 5-D itch scale is a reliable and multidimensional measure of uremic pruritus that is validated in hemodialysis patients. The 5-D itch scale categories are presented in this study based on its association with NRS categories. We purposed a transformation equation between the 5-D itch scale and NRS for pruritus measurements.

## Additional file

Additional file 1: Raw data. (XLSX 18 kb)

## Abbreviations

HD: Hemodialysis
NRS: Numerical rating scale
